# P-677. Differentially Expressed Genes in Patients with Legionella and Streptococcus Pneumonia

**DOI:** 10.1093/ofid/ofaf695.890

**Published:** 2026-01-11

**Authors:** Paulina Sudnik, Edward E Walsh, Ann R Falsey, Angela R Branche

**Affiliations:** University of Rochester, Rochester, NY; University of Rochester Medical Center, Rochester, NY; University of Rochester School of Medicine, Rochester, New York; University of Rochester, Rochester, NY

## Abstract

**Background:**

*Streptococcus pneumoniae (SP)* and *Legionella pneumophila (LP)* are important etiological pathogens of community-acquired pneumonia (CAP). Empiric treatment of CAP targeting typical organisms may be associated with worse outcomes in patients with Legionnaires' disease. We questioned whether differentially expressed genes (DEGs) in whole blood could discriminate between patients with streptococcal and legionella infections, which might impact current host-response diagnostics under development.

**Figure 1.**
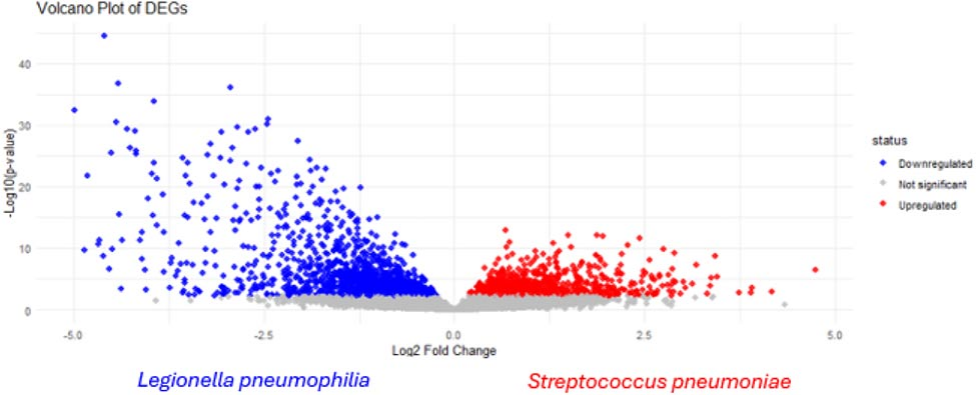
Volcano Plot of DEGs Streptococcus pneumoniae vs Legionella pneumophila. *DEGs with positive Log2 fold change marked in red illustrate genes relatively upregulated in patients with Streptococcus vs Legionella. DEGs with negative Log2 fold change marked in blue illustrate genes relatively upregulated in patients with Legionella vs Streptococcus.

**Figure 2.**
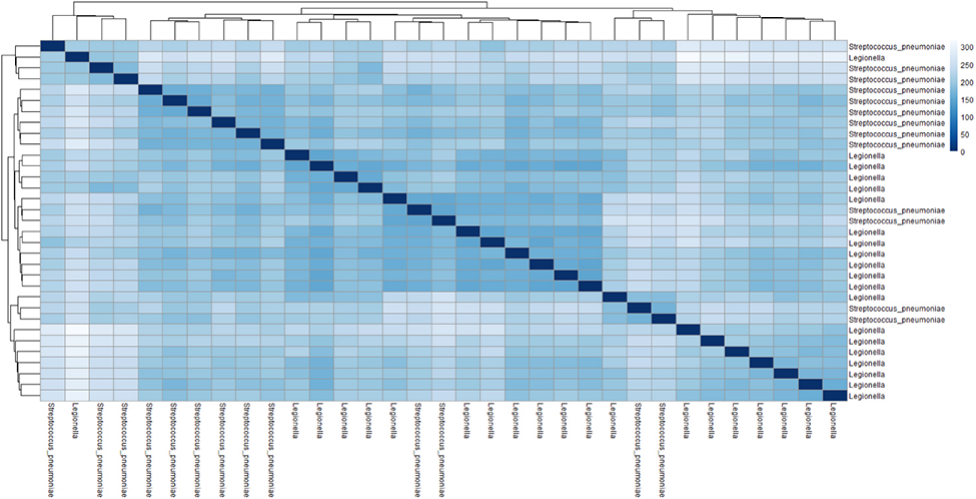
The Heatmap of Sample-to-Sample Distances. *The heatmap was constructed using Euclidian distance metrics. Darker colors indicate lower distances and higher similarity between samples. The dendrograms on the top and left sides of the heatmap illustrate the hierarchical clustering of the samples.

**Methods:**

This study used data prospectively collected from patients admitted with pneumonia from 2019 to 2021. Twenty patients with confirmed *LP* and 13 with *SP* had whole blood RNA-sequencing. DEGs between the samples were analyzed using RStudio packages "BiocManager", “dplyr”, “DESeq2”, “ggplot”, and “pheatmap”. DEGs were defined as those with an adjusted p-value cutoff of < 0.05. Functional enrichment analysis was conducted using the ToppFun tool to identify significant biological processes and pathways in genes.

**Results:**

A total of 2408 DEGs were identified, with 1031 genes upregulated in patients with *LP (*Figure 1). Genes activated in patients with LP were associated with an interferon activation pathway. The transcriptional profile reflected cellular processes related to cell membranes, vacuoles, and cytoplasmic transport. In patients with SP, the prostaglandin synthesis pathway was activated. Upregulated biological and cellular processes included inflammatory response, cell migration, cell-cell signaling, and kinase activity. Notably, the transcriptional signatures of patients with LP resembled influenza infection. The heatmap of sample-to-sample distances showed that most samples with the same pathogen clustered together (Figure 2). Confounding factors may have included underlying conditions and treatments such as steroid administration.

**Conclusion:**

Patients with *LP* and *SP* pneumonia have different gene expression patterns in whole blood. Differences in other intracellular pathogens, such as *Mycoplasma pneumoniae,* should be explored as they may affect the accuracy of host-related diagnostics.

**Disclosures:**

Edward E. Walsh, MD, Merck, Sharpe and Dohme: Advisor/Consultant|Merck, Sharpe and Dohme: Grant/Research Support|Moderna: Advisor/Consultant|Moderna: Grant/Research Support|Pfizer: Advisor/Consultant|Pfizer: Grant/Research Support|Sanofi: Advisor/Consultant|Sanofi: Honoraria Ann R. Falsey, MD, ADMA Biologics: Advisor/Consultant|ADMA Biologics: Honoraria|AstraZeneca: Advisor/Consultant|AstraZeneca: Grant/Research Support|AstraZeneca: Honoraria|CynaVac: Grant/Research Support|GSK: Advisor/Consultant|GSK: Honoraria|Merck: Advisor/Consultant|Merck: Honoraria|Moderna, Inc.: Advisor/Consultant|Moderna, Inc.: Grant/Research Support|Moderna, Inc.: Honoraria|Pfizer: Grant/Research Support|Sanofi Pasteur: Advisor/Consultant|Sanofi Pasteur: Honoraria Angela R. Branche, MD, Cyanvac: Grant/Research Support|Merck: Advisor/Consultant|Moderna: Advisor/Consultant|Moderna: Grant/Research Support|Pfizer: Grant/Research Support|Sanofi: Advisor/Consultant

